# Predicting neural activity of whole body cast shadow through object cast shadow in dynamic environments

**DOI:** 10.3389/fpsyg.2024.1149750

**Published:** 2024-04-05

**Authors:** Irini Giannopulu, Khai Lee, Elahe Abdi, Azadeh Noori-Hoshyar, Gaelle Brotto, Mathew Van Velsen, Tiffany Lin, Priya Gauchan, Jazmin Gorman, Giuseppa Indelicato

**Affiliations:** ^1^Creative Robotics Lab, UNSW, Sydney, NSW, Australia; ^2^Clinical Research and Technological Innovation Centre, RCIT, Paris, France; ^3^Department of Mechanical, Aerospace and Mechatronics Engineering, Monash University Australia, Melbourne, VIC, Australia; ^4^School of Engineering, Information Technology and Physical Sciences, Federation University, Ballarat, VIC, Australia; ^5^Interdisciplinary Centre for the Artificial Mind (iCAM), Gold Coast, QLD, Australia

**Keywords:** objects, body, shadow, substitution of the physical body, neural prediction, multiple regression analysis, EEG, VR immersion

## Abstract

Shadows, as all other objects that surround us, are incorporated into the body and extend the body mediating perceptual information. The current study investigates the hypothesis according to which the perception of object shadows would predict the perception of body shadows. 38 participants (19 males and 19 females) aged 23 years on average were immersed into a virtual reality environment and instructed to perceive and indicate the coincidence or non coincidence between the movement of a ball shadow with regard to ball movement on the one hand, and between their body shadow and their body position in space on the other. Their brain activity was recording via a 32-channel EEG system, in which beta (13.5–30 Hz) oscillations were analyzed. A series of Multiple Regression Analysis (MRA) revealed that the beta dynamic oscillations patterns of the bilateral occipito-parieto-frontal pathway associated with the perception of ball shadow appeared to be a significant predictor of the increase in beta oscillations across frontal areas related to the body shadow perception and the decrease in beta oscillations across frontal areas connected to the decision making of the body shadow. Taken together, the findings suggest that inferential thinking ability relative to body shadow would be reliably predicted from object shadows and that the bilateral beta oscillatory modulations would be indicative of the formation of predictive neural frontal assemblies, which encode and infer body shadow neural representation, that is, a substitution of the physical body.

## 1 Introduction

Whole body perception is considered an unconscious inference, albeit human beings are experts in representing and recognizing their body (Bubic et al., 2010). Humans exhibit an important variety of perceptual and motor behavior that enables them to interact with various objects within different environments. They can easily mentally represent the different parts of their body (Giannopulu and Mizutani, 2021), reconstruct body movements and immobility (Patel et al., 2022), predict the body’s future trajectory (Israël et al., 2013), and analyze and understand actions made by and with objects, including their shadow (Pavani and Galfano, 2015). Humans are skilled at analyzing and comprehending the shape, shadow, identity, and movement of objects, whether in real or virtual environments (Bonfiglioli et al., 2004; Lovell et al., 2009). Intriguingly, not only are the body and objects interwoven and incorporated (Yamamoto et al., 2005; Higuchi et al., 2007; Giannopulu et al., 2022a,b), and inherently predictive (Bays et al., 2006), but their shadows are tenuous components of the visual environment (Watanabe, 2018). Shadows prolong objects and the body beyond their physical boundaries (Kuylen et al., 2014). Considering an object’s shadow as an extension of the body from where the body shadow *per se* could be inferred, in the current study the bilateral electrical brain activity of healthy participants was recorded when immersed in a virtual environment. They were instructed to judge the coincidence or non coincidence between the movement of a ball and its shadow on the one hand and their body and its shadows on the other.

### 1.1 Theoretical background on objects and body shadows

Though watching an object’s static shadow facilitates object recognition (Elder et al., 2004), the moving object’s shadow, although omnipresent, usually appears to be misinterpreted with regard to one’s position (Kersten et al., 1997). Most studies have analyzed the role of motionless object shadows presented on a computer screen, and provided consistent and valuable information with regard to the visuospatial relationship between the objects’ shadows and the objects themselves (Madison et al., 2001). More specifically, they have demonstrated that shadows highly contribute to the accurate evaluation of object distance (Allen, 1999) but do not affect object recognition (Braje et al., 2000). The identification of geometric and familiar objects was found to be easier when they were presented with congruent rather than incongruent shadows (Castiello, 2001). In essence, shadows act unambiguously in affecting the visuospatial location of the objects casting them, while they ambiguously participate in recognizing the objects that cast them. Studies have also shown that a moving shadow influences the perceived motion of objects by inducing illusory motion in the depth of the objects (Kersten et al., 1996). At visuomotor performance level, when for instance, participants were reaching and grasping for a real object shadow visually presented, shadows specifically affected the kinematics and trajectory of movement execution (Bonfiglioli et al., 2004). Such results suggest that in object-oriented actions (i.e., reaching or grasping), shadows may participate in the planning and execution of the action as they represent supplemental features of the object. With respect to one’s own position, it appears that during motor performance, shadows would be intimately associated with the visuospatial system because they serve as the spatial scheme of a given environment (Kuylen et al., 2014). Moreover, when objects and shadows are in synchronized movement, they furnish relevant information with regard to the relationship between objects and shadows and specify the spatial arrangement of objects within an environment, i.e., they provide indications for the relative disposition of objects in space (Mamassian et al., 1998).

Contrary to the assumption that shadows are ignored or represented coarsely by the visual system (Rensink and Cavanagh, 2004), recent findings supported the idea that shadows are processed quickly and provide information about the properties of the environment (Lovell et al., 2009). de-Wit et al. (2012) examined the representational status of objects shadows when projected into the environment and reported that these shadows would emanate from region-based environmental segmentation instead of the representations of the objects *per se*. Interestingly, the brain refers and infers signals from body parts (e.g., the hand) directly to the object location (Paillard, 1991; Yamamoto et al., 2005), most likely because the connections between the hand and the object, including the object’s location, appear to be mentally represented and simulated (Parsons et al., 1995) and thus have neural correlates (Iriki et al., 1996, 2001; Higuchi et al., 2007; Katsuyama et al., 2016).

Objects are incorporated into the body (Giannopulu, 2016; Giannopulu et al., 2022a), are internalized and likewise they extend the body (Maravita and Iriki, 2004; De Preester and Tsakiris, 2009). With regard to one’s position, because internalized, object shadows are both objects and body extensions (Kuylen et al., 2014). As part of the object, object shadows elongate the object beyond its limits, while body shadows expand the body outside its corporal entity (Kuylen et al., 2014; Kodaka and Kanazawa, 2017; Hirakawa et al., 2020). Body shadows project images of the body in the environment, appear to have structural and anatomical similitudes with the body parts (Pavani and Galfano, 2007), and are subsequently synchronized to body motion. In both real and virtual environments, studies suggested that body shadows might enrich the representation of body position in space by strengthening the relation and interaction with the objects (Pavani and Castiello, 2004; Russo et al., 2017). It was also demonstrated that in the absence of any object feedback, visual or tactile, object processing is used as support for body shadows (Kuylen et al., 2014). Nevertheless, it is unclear whether the perception of object shadows would be associated with the perception of body shadows. However, if an object’s shadows are extensions of the body and body shadows are also extensions of the body, the perception of object shadows would predict the perception of body shadows. Thus, by incorporating both the object shadow and body shadow, as well as their relationship, it can be expected that inference and prediction of body shadow, which are inherently associated with neural processing, would enable humans to process and perform decisions accurately and quickly. Consequently, the investigation of brain activity related to object shadow with inferential and predictive mechanisms to the brain activity of body shadow was performed.

### 1.2 Neural support for predicting the relationship between object and body shadows

Poirier and Hardy-Vallee (2005) suggested that the brain emulates the body and simulates the external events (e.g., objects) based on sensorimotor representations. Both simulations and emulations behave as internal models, and are predictive in essence (Bays et al., 2006). Such models may be considered in order to estimate the current state or envision the future state of the nervous system (Miall and Wolpert, 1996; Wolpert and Flanagan, 2001). Deciphering the neural implementation of body representations, Bubic et al. (2010) reported that the predictive processing is associated with a series of neural networks that includes cortico-cortical (i.e., occipital, parietal, frontal and prefrontal) and sub-cortical areas (i.e., thalamus, cerebellum, basal ganglia). Even though neocortical beta oscillations (15–29 Hz) are strong indicators of perceptual performances in humans (Sherman et al., 2016), predictive processing modulates sensory cortices (Gómez et al., 2004). Anterior brain areas, such as frontal and prefrontal, were considered as bases which specify, prepare and plan intentions and communicate them to sensory areas (Bubic et al., 2010). Nevertheless, it is conceivable to assume that a more unified neural network, signifying neural synchronization/desynchronization between relevant cortical areas, would be a potential indicator of object-body shadow predictive processing. Notwithstanding, when line drawing of hands, real hand actions and intransitive movements by hand cast shadow were observed, significant desynchronization of mu activity (8–13 Hz) across sensorimotor, frontal and central and right parietal cortices relative to the baseline was revealed, but neither a relationship between them nor difference in mu activity was reported in all cases (Zhu et al., 2013). The observation of shadow animations depicting a figure’s motion, that is, recognition of biological motion, showed specific resonance motor responses in M1 (Alaerts et al., 2009), in the bilateral MT (Katsuyama et al., 2016) and also involved mirror neurons system activity (Sartori and Castiello, 2013). At a more general functional and behavioral level, patients with neglect resulting from frontal and parietal lesions were able to perceive shadows explicitly or implicitly (Castiello et al., 2003) regardless of their spatial location (left or right of the object). More importantly, in virtual reality environments, body shadow appears to positively contribute to neurocognitive motor improvement after prefrontal, frontal and parietal brain damages (Russo et al., 2017). However, little is known about the perception of object shadows and their relation with the body shadow in virtual reality environments. The aim of the current study is also to fill this gap.

Immersed in a virtual reality environment, healthy participants were instructed to perceive and indicate the coincidence or non coincidence between a mobile ball shadow with regard to a mobile ball from the one side, and the coincidence or non coincidence between their own body shadow and position in space from the other. Their brain activity was recorded using a 32-Channel Wireless EEG system. Given that object and body shadows involve left and right hemispherical activity as already reported, bilateral beta (13.5–30 Hz) oscillations dynamics of frontal, parietal and occipital brain areas were analyzed as they are considered predictors of perceptual and motor performance (Sherman et al., 2016). Taking into consideration the above analyzed arguments and referred studies, it was hypothesized that beta oscillations associated with the perception of a ball shadow would envision the neural activity of the body shadow. It was expected that the fronto-patieto-occipital beta neural oscillations associated with the perception of an object shadow would predict the neural activity (synchronization vs. desynchronization) of the body shadow.

## 2 Method

### 2.1 Participants

The feasibility of the study was assessed via an *a priori* G*Power 3.1. The results have shown that the minimum number of participants required was 36 in order to achieve an adequate statistical power of 0.85 with a medium effect size (*d* = 0.30), and alpha level of 0.05. Forty participants were recruited for the study, consisting of twenty males and twenty females with an average age of 23.63. Their right-handedness was about 100% according to the Edinburgh Handedness Inventory (Oldfield, 1971). All the participants were from a middle to high socioeconomic background and none had specific training experience with virtual reality environments. The participants had normal or corrected-to-normal vision and declared that they were free of vestibular, cardiac or sensorimotor and/or neurological disorders. All participants received a $50 gift card upon completion of the study. The participants had average somatosensory performances as assessed by the Rivermead Assessment of Somatosensory of Performance (RASP) (Winward et al., 2000). The final sample consisted of only 38 participants (1 participant experienced motion sickness, and 1 was eliminated for technical reasons). Approval was granted by the University Human Research Ethics Committee (BUHREC 16121) and conformed to the declaration of Helsinki 2.0. Informed consent for study participation was required and obtained from all participants. Anonymity was guaranteed.

### 2.2 Experimental setup

#### 2.2.1 EEG device

The electrical brain activity of the participants was recorded using a Mobita 32-Channel Wireless EEG System (Biopac Systems Inc.). The international 10/20 extended system was used in order to equally distribute all the electrodes over the scalp: Fp1, Fpz, Fp2, F7, F3, Fz, F4, F8, FC5, FC1, FC2, FC6, T7, C3, Cz, C4, T8, TP9, CP5, CP1, CP2, CP6, TP10, P7, P3, Pz, P4, P8, PO, O1, Oz, and O2. The right clavicle was used for the reference electrode; the sampling rate was set at 1,000 Hz (Patel et al., 2022; Giannopulu et al., 2022a,b; Giannopulu et al., 2023). Mobita’s quality and reliability are given by Bateson et al. (2017).

#### 2.2.2 Head-mounted device

The HTC Vive used in the study was a system consisted of a 2,160 × 1,200 resolution headset including a front camera and adjustable straps. It also had two sensors with SteamVR Tracking 1.0 technology and two motion-tracked controllers. Using the sensors a 360 degrees virtual environment (3.5 m x 3.5 m) can be created. A computer with the minimum requirements to operate the HTC Virtual Reality system software (Vive, 2011) was used. More particularly, the VR program ran on the DELL Precision 5,820 computer with Windows 10 programming, Intel Core Xeon 4 processing system, 32 GB RAM, HDMI 1.4 port and GeForce GTX 970 graphics card.

#### 2.2.3 Virtual reality environment

**Figure 1:**
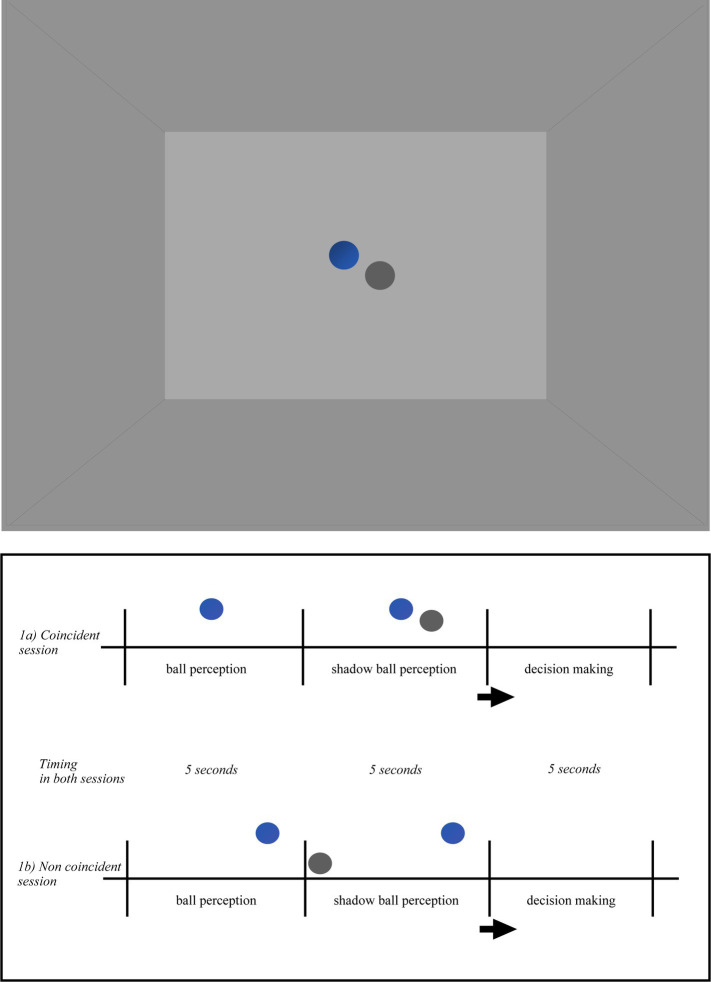
Pictorial illustration of the virtual environment in the ball shadow condition (BaSC). Ball and shadow trajectories were always presented within the horizontal visual field of the participant and arranged according to the following scenarios: (i) the ball first (5 s) and the shadow after (5 s) were descending toward the floor or ascending toward the ceiling (vertical axis); (ii) the ball first (5 s) and the shadow after (5 s) were moving forward on the anterior–posterior axis across the floor (parallel/sagittal axis); (iii) the ball first (5 s) and the shadow after (5 s) were moving on the lateral axis toward the left or toward right side (lateral/parallel to the ground); (iv) the ball first (5 s) and the shadow after (5 s) were moving diagonally upwards or downwards (diagonal axis). The order of the trajectories of the ball and shadow was randomized across the participants. When the movement between the ball and its shadow was coincident (1a), both described the same linear (sagittal, vertical, lateral, or diagonal) trajectory. When the movement between the ball and its shadow was non coincident (1b) the ball trajectory was the same as above, but the ball shadow followed a different trajectory than the ball. Taking as reference their own position in space, the participants were told to indicate whether the movement of the ball shadow (i.e., with shadow) was coincident or not with the movement of the ball.

**Figure 2:**
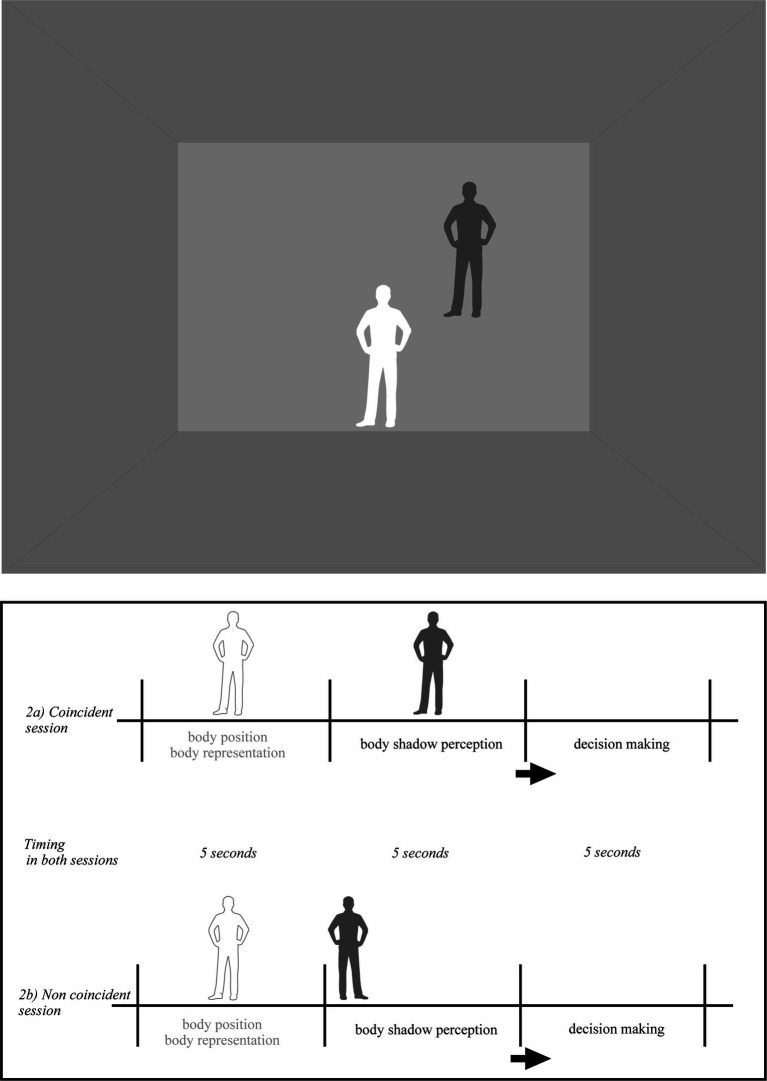
Pictorial illustration of the virtual environment in the body shadow condition (BoSC). The shadow of each participant appeared on the frontal plan. Body shadow scenarios were as follows: (i) without body shadow first (5 s) and with body shadow after (5 s) where the shadow was ascending toward the wall (vertical axis); (ii) without shadow first (5 s) and with body shadow after (5 s) where the shadow was moving forward on the anterior–posterior axis across the floor and projected onto the wall (sagittal axis); (iii) without shadow first (5 s) and with body shadow after (5 s) where the shadow was moving on the lateral axis toward the left or toward right side on the wall (lateral/parallel to the ground). The order of body and shadow was randomized across the participants. When the body and its shadow coincided (2a), the shadow of the participant’s body projected onto the frontal plane matched the position of the participant’s body. When the body and its shadow were not aligned (2b), the shadow cast on the frontal plane did not correspond to the position of the participant’s body. The participants were told that they had to take their own position as reference, and decide if the shadow was coincident or non coincident (i.e., conforming or non conforming) with the position of their body in space.

All participants were immersed in a Virtual Reality Environment, in a cubic room specially designed for the study. The room consisted of three walls (one front and two side), a ceiling and a floor. The room was empty. The two lateral walls, the floor and the ceiling were colored in grey, the frontal wall was colored in pale grey. The colors and light in the room were constant during the whole experiment. Once equipped with the EEG, the Head-mounted Device (HMD) and the controllers (i.e., left and right key-response), the participants were fully immersed in the room (Figures 1, 2). Following the experimental condition, a ball and its shadow or each participant’s body shadow appeared always at the same distance from the participant’s spatial position. There were two independent conditions: ball shadow condition (BaSC) and body shadow condition (BoSC).

In the BaSC condition (as shown in Figure 1), a spherical ball and its shadow move either coincidentally or non coincidentally. The incident light to the ball was parallel, the shadow silhouette was cast on the horizontal projection plane. The ball was opaque, its shadow was solid. The color of the ball was blue, the color of its shadow was dark-grey. The shape, size and distance between the ball and shadow were constant across the participants in both coincident and non coincident sessions. The speed of the ball and its shadow was slow (translational speed 4 cm/s, angular speed 8.5 deg./s) and constant, their trajectory was linear along the vertical, lateral or diagonal axis within the horizontal plane. Ball and shadow trajectories were always presented within the horizontal visual field of the participant and arranged according to the following scenarios: (i) the ball first (5 s) and the shadow after (5 s) were descending toward the floor or ascending toward the ceiling (vertical axis); (ii) the ball first (5 s) and the shadow after (5 s) were moving forward on the anterior–posterior axis across the floor (parallel/sagittal axis); (iii) the ball first (5 s) and the shadow after (5 s) were moving on the lateral axis toward the left or toward the right side (lateral/parallel to the ground); (iv) the ball first (5 s) and the shadow after (5 s) were moving diagonally upwards or downwards (diagonal axis). The order of the trajectories of the ball and shadow was randomized across the participants. When the movement between the ball and its shadow was coincident (Figure 1a), both followed a linear (sagittal, vertical, lateral or diagonal) trajectory. On the contrary, when the movement between the ball and its shadow was not coincident (Figure 1b) the ball trajectory was the same as above, but the ball shadow followed a different linear trajectory to the ball.

In the BoSC condition (Figure 2), the shadow of each participant was the same color (dark-grey) as previously described and appeared in a coincident or non coincident position with regard to its body position in space. The incident light to the participants body was parallel, its shadow silhouette was cast on the horizontal projection plane and was moving linearly at a constant speed starting from the ground and projected into the front wall. Its speed was linear and constant (as in the BaSC condition). The body shadow scenarios were the following: (i) without body shadow first (5 s) and with body shadow after (5 s) where the shadow was ascending toward the wall (vertical axis); (ii) without shadow first (5 s) and with body shadow after (5 s) where the shadow was moving forward on the anterior–posterior axis across the floor and projected on the wall (sagittal axis); (iii) without shadow first (5 s) and with body shadow after (5 s) where the shadow was moving on a lateral axis toward the left or right side of the wall (lateral/parallel to the ground). As previously, the order of body shadow was randomized across the participants. When the body and its shadow were coincident (Figure 2a), the body shadow projected onto the frontal plane of body participant (i.e., front wall of the room) was consistent with the body position of each participant in space. When the relationship between the body and its shadow was non coincident (Figure 2b), their shadow projected onto the frontal plane and was inconsistent with the participants body position in space.

### 2.3 Procedure

The experiment consisted of three phases: *baseline, initiation and experimental phase*. All three phases took place in the same dark and quiet experimental room. The inter phase interval was approximately 3 min.

The *baseline* consisted of one-minute EEG recording in the dark while participants were in the experimental room remaining speechless and motionless.

During *the initiation phase*, participants were given five trials in two different conditions independently: the ball shadow condition (BaSC), and the body shadow condition (BoSC). Half of the participants started with the BaSC, and the other half with the BoSC in a randomized order. In the BaSC condition, all participants were placed in the same position and it was explained that they had to look straight ahead in front of them without moving their head, body or arms. They were also instructed that a ball in movement would appear (i.e., without shadow) and a ball shadow (i.e., with shadow) would also appear within their horizontal plane. Taking as reference their own position in space, the participants were told to indicate whether the movement of the ball shadow (i.e., with shadow) was coincident or not with the movement of the ball. They were also instructed to click on a left key-response in the former case (i.e., coincident); and on a right key-response in the latter (i.e., non coincident) as quick as possible. The participants were allowed 5 s to take a decision (i.e., decision making). The order of the ball and shadow was randomized across all participants.

In the BoSC condition, the participants were instructed to look straight ahead without moving as previously (i.e., without shadow). They were also instructed that the shadow of their body would appear (i.e., with shadow) within their horizontal plane. They were told that they had to take their own position as reference, and decide if the shadow was coincident or non coincident (i.e., conforming or non conforming) with the position of their body in space once the shadow was ceased moving onto the front plane. As in the BaSC condition, the participants were given 5 s to produce a response (i.e., decision making) as quick as possible. The order of the body shadow was randomized across all participants.

The inter trail interval was approximately 15 s; and the inter condition interval was approximately 3 min. According to the criteria, only participants who provided three correct consecutive trials in each condition (BaSC and BoSC) and declared themselves not to experience motion sickness were included in the experimental phase.

During the *experimental phase*, the participants were immersed in the same virtual environment as in the initiation phase. They were placed in the same spatial location as previously and were again instructed to look straight ahead and remain motionless. All participants were given 3 min in BaSC, and 3 min in BoSC condition. Half of the participants started with the BoSC condition, and the other half with the BaSC condition. The inter condition interval was approximately 3 min.

In the BaSC condition (Figures 1a,b), the sequence of events was exactly the same as in the initiation phase, and was the following: without shadow (i.e., ball movement for 5 s), with shadow (i.e., ball and shadow in movement for 5 s), and decision making (i.e., 5 s). Once again the participants were instructed to use their own position as a reference and to indicate if the movement of the ball shadow (BaSC) was coincident (press left key-response) or not coincident (press right key-response) with the movement of the ball as fast as possible. As previously, the order of the trajectories of the ball and shadow was randomized across the participants.

Likewise, in the BoSC condition (Figures 2a,b), the sequence of the events for the participants was: without shadow (i.e., body shadow to appear 5 s after); with shadow (i.e., 5 s for the body shadow in movement); and decision making (i.e., 5 s) once the shadow was immobilized onto the frontal space. According to the instructions, the participants had to press the left key-response, if the shadow of their body was coincident with the position of their body in space, and the right key-response if it was not coincident as fast as possible. Once again and for methodological reasons (i.e., control for order effects), the order of the body shadow was randomized across all participants.

In both BaSC and BoSC conditions, the brain activity of the participants was recorded continuously via the 32-Channel EEG system. In addition, participants’ reaction time (RT) was automatically recorded during the decision making session. The RT corresponded to the duration of time between the shadow apparition (ball or body shadow) and the pressing of the key-response and was measured in milliseconds (ms). The total procedure lasted about 45 min on average.

### 2.4 Data analysis

#### 2.4.1 EEG signal processing and preprocessing

EEG data was preprocessed and processed with MATLAB (Version R2020b) and FieldTrip toolbox (Oostenveld et al., 2011). Only the data of the experimental phase was considered for all trials and participants. Specifically, 5 s associated with the presence (i.e., with), the absence (i.e., without) of the shadow and the decision making session within each condition (i.e., BaSC vs. BoSC) were examined. Each “with,” “without” and “decision making” 5 s event was marked at the onset and the end for each trial and participant with a buffering of 20 ms before and after each 5 s period for each experimental condition (i.e., BaSC and BoSC) and a baseline correction of −10 to −30 ms. A high-pass filter of 1 Hz and a low-pass filter of 40 Hz composed the preprocessing and processing script. Artifact detection was performed on all marked events. First all bad channels and high-amplitude EEG artifacts, i.e., above 30 microvolts, were automatically removed from all events. Then all additional artifacts including electromyogram, electrooculogram and electrocardiogram were eliminated manually after visual inspection by experts and corrected via independent component analysis (ICA) methods. To ensure data quality, all data was again visually inspected by two independent experts and the remaining artefacted events were manually removed blind to the experimental condition (i.e., BaSC and BoSC) and 5 s events (i.e., “with,” “without” and “decision making”). 94% of the trials were preserved, while 3.1% of trials with EOG artifacts and 2.9% of trials with EMG artefacted events were eliminated. The processing script performed a beta frequency analysis (13.5–30 Hz) on all filtered 5 s events per experimental condition. The frequency analysis resulted in an average power spectral density measured in microvolts per Hertz (mV^2^/Hz) in frontal, parietal and occipital areas for beta oscillations in both left and right hemispheres (i.e., bilateral beta oscillations dynamics). The 32 electrodes were grouped into 3 regions of interest (ROIs) in order to effectively cover the difference cortical regions bilaterally (i.e., both left and right hemispheres) of the brain. The analogy between each ROIs and electrodes was: left and right frontal (F7, F3, Fz, F4, F8, FC5, FC1, FC2, FC6, C3, Cz, C4), left and right parietal (CP5, CP1, CP2, CP6, P7, P3, Pz, P4, P8), and left and right occipital (PO, O1, Oz, O2) areas. Although a large number of electrodes design are possible, the aforementioned bilateral design was selected as it covers all the brain areas and it is directly associated with the purposes of the present study. The statistical analysis was performed on the aforementioned marked and cleaned events of the experimental data (i.e., 912 trials for 38 participants).

### 2.5 Statistical analysis

All 38 participants successfully gave three correct consecutive trials in each condition (BaSC vs. BoSC), and did not declare motion sickness during the initiation phase and passed in the experimental phase. Only the experimental data was considered for the statistical analysis. Statistical analysis was completed in SPSS software package version 26.0.

A MANOVA was run to examine the effect of gender (i.e., female *vs* male), presence vs. absence [i.e., “with” (5 s) vs. “without” (5 s)] of shadow and the two dimensions of shadow occurrence [i.e., coincidence (5 s) vs. non coincidence (5 s)] between the shadow and the object on the bilateral beta frontal, parietal and occipital oscillations for the BaSC condition on the one hand, and the BoSC condition on the other, independently. The MANOVA was assessed at a 95% confidence level using Wilks’ lambda (λ) with a significance level of a = 0.05.

An one-way ANOVA was performed to analyze the effect of the two dimensions of shadow coincidence (i.e., 5 s vs. non coincidence, 5 s) on the reaction time in both BaSC and BoSC condition, independently.

A series of Multiple Regression Analysis (MRA) were performed to assess whether the bilateral neural activity in shadow perception (i.e., with shadow, 5 s) or decision making (i.e., coincident or non coincident, 5 s) in the body shadow condition (BoSC) would be inferred by the bilateral neural activity in the perception (i.e., with shadow, 5 s) or decision making (i.e., coincident or non coincident, 5 s) in the ball shadow condition (BaSC). Prior to performing the above comparisons and multiple regression analyses, several assumptions were verified. First, visual inspection of histograms: Shapiro Wilks (*p* > 0.05) and boxplots indicated that each variable in each comparison and regression was approximately normally distributed. One extreme outlier was removed while the other was kept as it did not affect the results. Second, the assumptions of normality, linearity, and homoscedasticity of residuals were met by inspecting the normal probability plot of standardized residuals as well as the scatterplot of standardized residuals against standardized predicted values for each MRA. Third, Mahalanobis distance did not exceed the critical χ^2^ for df = 3 (at α = 0.001) of 16.27 for all cases in the data file, indicating that multivariate outliers were not of concern. Fourth, relatively high tolerances for each predictor in the regression model indicated that multicollinearity would not interfere with the ability to interpret the outcome of the multiple regression analyses. All statistical analyses were performed at alpha 0.05.

## 3 Results

All the participants correctly assessed the relationship between the ball and its shadow on the one hand and their body position and body shadow on the other (i.e., overall error rate = 0% in both experimental conditions). No gender effect has been observed (Wilks’ lambda = 34.51, *F* = 8.92, df (2,78), *p* = 0.961).

### 3.1 With shadow vs. without shadow; coincidence vs. non coincidence of responses in decision making

In BaSC condition, the multivariate results were not significant for ball shadow presence vs. absence (i.e., with vs. without), Wilks’ lambda = 10.31, *F* = 5.52, *df* = (2,78), *p* = 0.886, and ball shadow occurrence (i.e., coincident vs. non coincident) Wilks’ lambda = 21.34, *F* = 1.52, *df* = (2,66) *p* = 0.699 on the bilateral beta frontal, parietal and occipital oscillations.

Similarly, in the BoSC condition, no significant multivariate results were found for body shadow presence vs. absence (with vs. without) Wilks’ lambda = 9.12, *F* = 2.39, *df* = (2,78), *p* = 0.485, and body shadow occurrence (i.e., coincident vs. non coincident) Wilks’ lambda = 31.79, *F* = 7.88, *df* = (2,66) *p =* 0.683 on the bilateral beta oscillations (frontal, parietal and occipital).

Overall, the results imply that the perception of the ball or the ball shadow in the BaSC condition on the one hand, and the body or the body shadow in the BoSC on the other involved similar anterior and posterior brain activities at beta oscillation level bilaterally. Additionally, and in both shadow conditions, that is, within BaSC and within BoSC, the coincidence or non coincidence between the movement of the ball and its shadow, on the one hand, and the body and its shadow, on the other, did not affect neural activity differently.

### 3.2 Reaction time in ms (RTs)

In BaSC condition, the one-way ANOVA showed that there was no statistically significant difference in reaction time (RTs) between the two dimensions of shadow occurrence (i.e., coincident vs. non coincident) (mean = 1,254 ms, sd = 74 for coincident vs. mean = 1,218 ms, sd = 97 for non coincident; *F*(2, 36) = 1.13, *p* = 0.714).

Regarding the BoSC condition, the one-way ANOVA did not reveal significant difference in reaction time (RTs) when the body and its shadow were coincident and when they were not coincident (mean = 993 ms, sd = 69 for coincident and mean = 1,021 ms, sd = 53, for non coincident; *F*(2, 36) = 0.73, *p* = 0.599).

Specifically, in both independently evaluated conditions, the reporting time of coincidence or non coincidence (i.e., RTs) between the movement of the ball and its shadow (BaSC) on the one hand, and the position of then body and its shadow (i.e., BoSC) on the other was similar.

### 3.3 Ball shadow as a predictor of body shadow

A series of MRA was run in order to determine if the bilateral neural activity of the perception and/or decision making of the body shadow (i.e., BoSC condition) would be predicted by the perception and/or decision making of the ball shadow (i.e., BaSC condition). Two MRAs were found to be significant.

**Figure 3:**
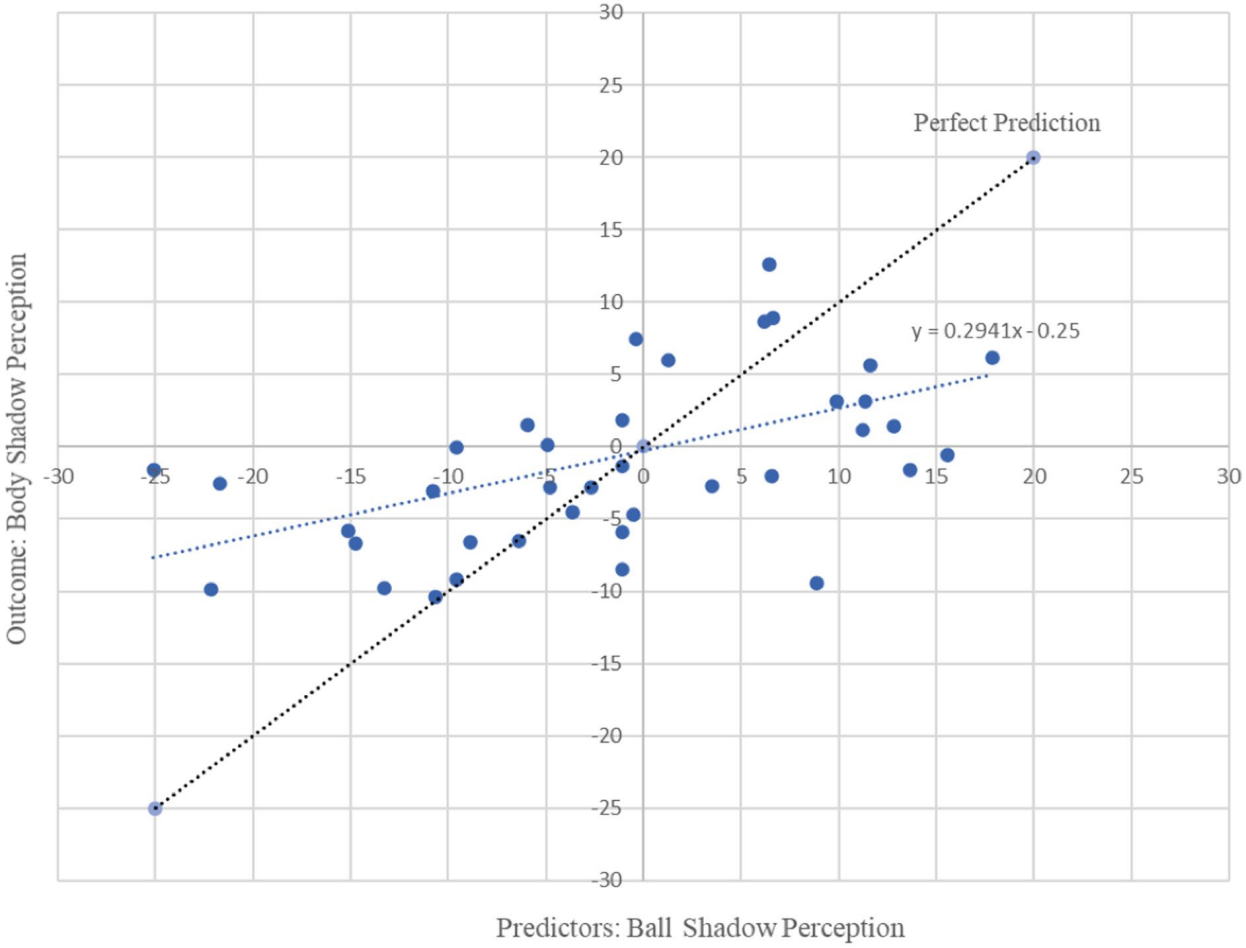
Graphical representation of multiple regression analysis (MRA) on Ball Shadow Perception (predictors) with regard to Body Shadow perception (outcome). X axis represents the unstandardized predicted value of each participant for all cerebral regions in combination (i.e., frontal, parietal and occipital) in the ball shadow perception; Y axis depicts the outcome of the prediction for each participant with respect to the Body Shadow perception (Frontal). Bilateral beta (13.5 to 30 Hz) oscillations of the frontal, parietal and occipital brain areas associated with ball shadow perception predicted the activation of frontal beta oscillations corresponding to the body shadow perception [*R*^2^ = 0.29, adjusted R^2^ = 0.22, *F*(3, 35) = 4.54, *p* = 0.009 combined effect of this magnitude can be considered “large” (f^2^ = 0.41)].

In combination the left and right beta oscillations (13.5 to 30 Hz) in frontal, parietal and occipital areas associated with the beta oscillations of the ball shadow (BaSC) were associated with body shadow perception (BoSC) (R^2^ = 0.29, adjusted R^2^ = 0.22, *F*(3, 35) = 4.54, *p* = 0.009). By Cohen (1988) conventions, a combined effect of this magnitude can be considered “large” (f^2^ = 0.41). As illustrated in Table 1, beta oscillations across sensorimotor frontal, parietal and occipital areas associated with ball shadow perception were significantly predictive of the future activation of bilateral frontal beta oscillations associated with body shadow perception (*B* = 1.09, *p* = 0.024; *B* = 0.21, *p* = 0.028; *B* = 0.91, *p* = 0.002 respectively). Specifically, the bilateral beta oscillations of frontal, parietal and occipital areas associated with the ball shadow perception were significant predictors of the activation of the bilateral frontal beta oscillations corresponding to the body shadow perception (Figure 3). However, bilateral frontal, parietal and occipital beta oscillations associated with ball shadow perception did not significantly predict the bilateral occipital (*p* = 0.369) and parietal (*p* = 0.467) neural activation associated with body shadow perception.

**Table 1:** Multiple regression analysis (MRA) on ball shadow perception (predictors) with regard to body shadow perception (outcome).

Body shadow perception Outcomes	Ball shadow perception Predictors			*B*	SE B	95% CI for B	*β*
Frontal	Constant	−0.25	2.60		
	Frontal	1.09*	0.46	[0.16; 2.02]	1.02
	Occipital	0.21*	0.09	[0.03; 0.40]	0.64
	Parietal	0.91**	0.28	[0.35; 1.48]	1.17
Occipital	Constant	0.82	4.14		
	Frontal	−1.87	1.18	[−4.28; 0.53]	−0.78
	Occipital	−0.29	0.24	[−0.78; 0.21]	−0.38
	Parietal	−1.35	0.71	[−2.80; 0.09]	−0.77
Parietal	Constant	−0.66	1.47		
	Frontal	−0.57	0.42	[−1.43; 0.29]	−0.66
	Occipital	−0.14	0.09	[−0.31; 0.04]	−0.51
	Parietal	−0.33	0.26	[−0.84; 0.19]	−0.52

In combination, beta oscillations (13.5 to 30 Hz) across the bilateral frontal, parietal and occipital areas related with ball shadow perception were significantly predictive of the future activation of bilateral frontal beta oscillations associated with the body shadow perception. However, bilateral frontal, parietal and occipital beta oscillations associated with ball shadow perception did not significantly predict the unilateral or bilateral occipital and parietal neural activation associated with body shadow perception.

**Figure 4:**
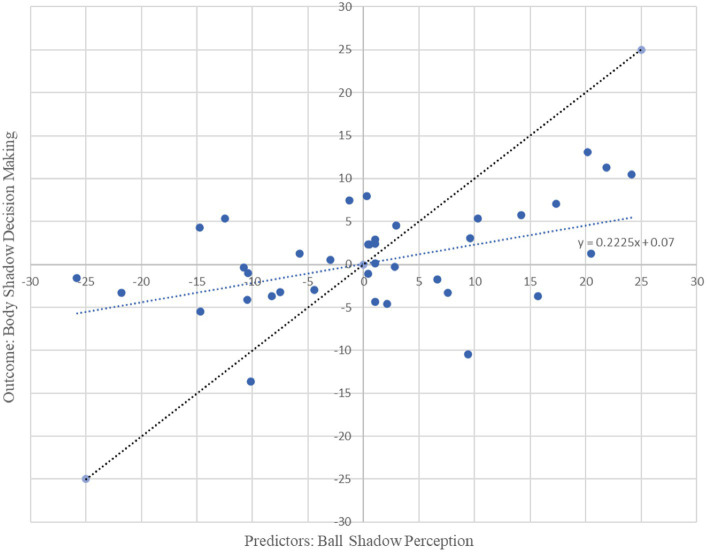
Graphical representation of multiple regression analysis (MRA) on Ball Shadow Perception (predictors) related to the decision making on body shadow (outcome). X axis represents the unstandardized predicted value of each participant for all cerebral regions pulled together (frontal, parietal and occipital) in the Ball Shadow Perception; Y axis depicts the outcome of the prediction for each participant with respect to the Body Decision making (Frontal). A decrease of bilateral beta frontal oscillations (13.5 to 30 Hz) related to decision making of the body shadow was predicted by a desynchronization of beta oscillations in frontal, parietal and occipital neural activity associated with ball shadow perception [*R*^2^ = 0.22, adjusted *R*^2^ = 0.15, *F*(3, 34) = 3.19, *p* = 0.036; combined effect of this magnitude can be considered “medium” (f^2^ = 0.28)].

Moreover, beta oscillations of the bilateral frontal, parietal and occipital neural activity associated with the ball shadow perception significantly accounted for 22% of the variability in the bilateral frontal neural activity of the decision making on body shadow (R^2^ = 0.22, adjusted R^2^ = 0.15, *F*(3, 34) = 3.19, *p* = 0.036). By Cohen (1988) conventions, a combined effect of this magnitude can be considered “medium” (f^2^ = 0.28). The beta oscillations (13.5 to 30 Hz) across sensorimotor frontal, parietal and occipital areas related to the ball shadow perception showed negative predictive values of the bilateral beta oscillations of frontal activity associated with the decision making related to the body shadow (*B* = −1.27, *p* = 0.022; *B* = −0.32, *p* = 0.005; *B* = −0.80, *p* = 0.018 respectively) (Table 2). In other words, the decrease of the bilateral beta frontal oscillations related to decision making concerning the body shadow was predicted by a bilateral desynchronization of beta oscillations in frontal, parietal and occipital neural activity associated with ball shadow perception (Figure 4). Nevertheless, the bilateral neural beta oscillations of frontal, parietal and occipital areas associated with the perception of the ball shadow did not significantly predict the bilateral parietal (*p* = 0.646) and occipital (*p* = 0.996) activity related to the decision making concerning the body shadow.

**Table 2:** Multiple regression analysis (MRA) on ball shadow perception (predictors) related to the decision making on body shadow (outcome).

Decision making on body shadow Outcomes	Ball shadow perception Predictions			*B*	SE B	95% CI for *B*	*β*
Frontal	Constant	0.07	1.84		
	Frontal	−1.27*	0.53	[−2.34; −0.20]	−1.08
	Occipital	−0.32**	0.11	[−0.54; −0.10]	−0.87
	Parietal	−0.80*	0.32	[−1.44; −0.15]	−0.93
Occipital	Constant	1.17	4.46		
	Frontal	0.87	1.27	[−1.72; 3.46]	0.38
	Occipital	0.26	0.26	[−0.28; 0.79]	0.32
	Parietal	0.38	0.77	[−1.17; 1.94]	0.62
Parietal	Constant	−0.66	1.47		
	Frontal	−0.57	0.42	[−1.43; 0.29]	−0.66
	Occipital	−0.14	0.09	[−0.31; 0.04]	−0.51
	Parietal	−0.33	0.26	[−0.84; 0.19]	−0.52

In combination, beta oscillations (13.5 to 30 Hz) across the bilateral frontal, parietal and occipital areas related to ball shadow perception showed negative predictive values of the beta oscillations of bilateral frontal activity associated with the decision making of the body shadow. Nevertheless, the neural beta oscillations of frontal, parietal and occipital areas associated with the perception of the ball shadow did not significantly predict the unilateral or bilateral parietal and occipital activity related to the decision making of the body shadow.

**Figure 5:**
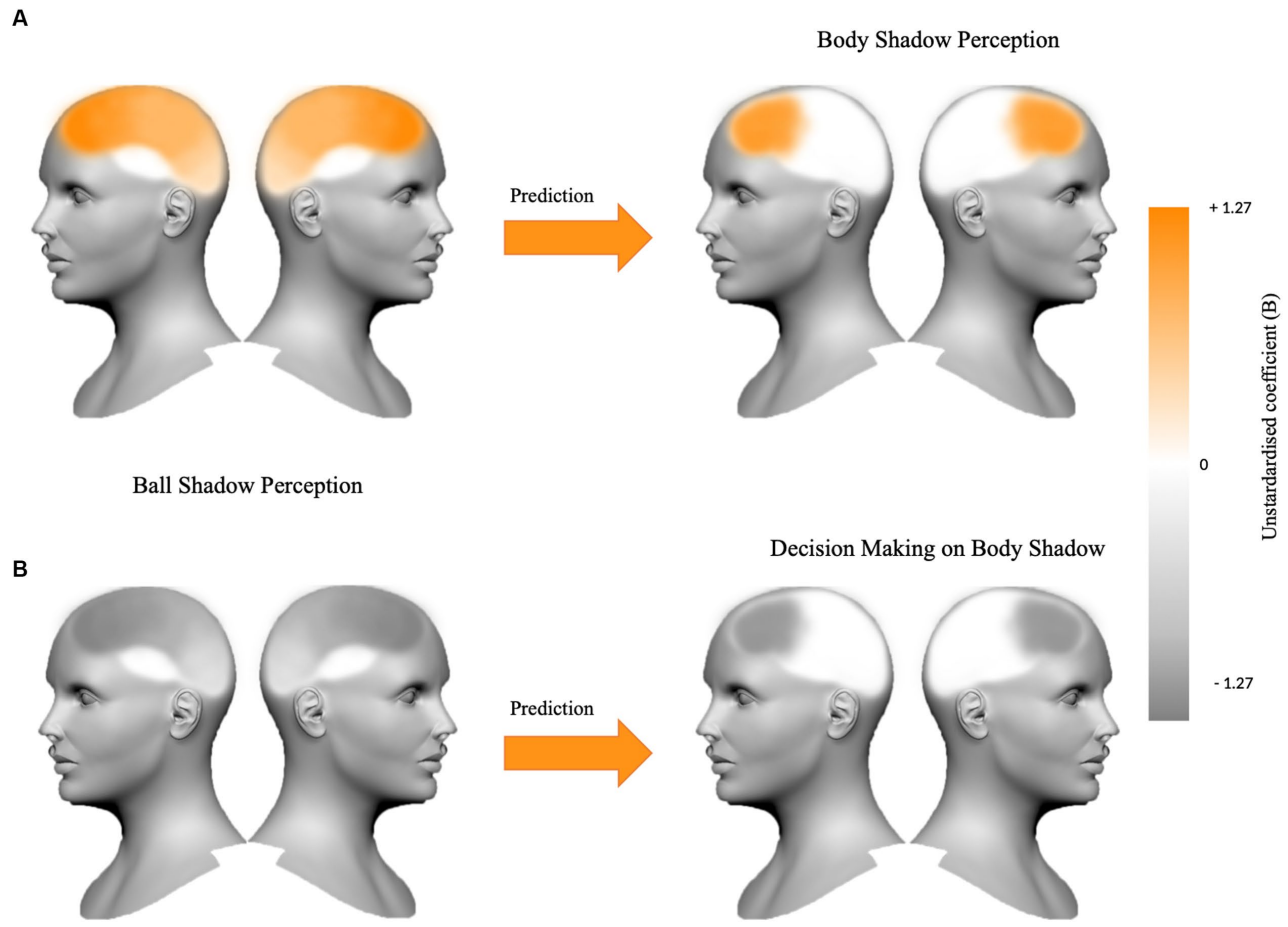
The figure summarizes **(A)** the beta oscillations in frontal, parietal and occipital areas bilaterally for ball shadow perception as a predictor of the increased frontal activity during body shadow perception (*B* = 1.09, *p* = 0.024; *B* = 0.21, *p* = 0.028; *B* = 0.91, *p* = 0.002 respectively), and **(B)** the bilateral decreased beta oscillations in frontal, parietal and occipital areas anticipating decrease frontal activity during the decision making on body shadow (*B* = −1.27, *p* = 0.022; *B* = −0.32, *p* = 0.005; *B* = −0.80, *p* = 0.018 respectively). The variations (+1.27 vs. −1.27) of the unstandardized coefficient (B) are illustrated on the color bar.

In summary, it appears that the synchronization of bilateral beta oscillations in anterior and posterior areas (i.e., frontal, parietal and occipital areas) during ball shadow perception (BaSC condition) predicted increased bilateral frontal activity during body shadow perception (BoSC condition). On the contrary, decreased bilateral beta oscillations in frontal, parietal and occipital areas of ball shadow perception (BaSC condition) were significant predictors of decreased bilateral frontal activity during the decision making on body shadow (BoSC condition) (Figure 5).

## 4 Discussion

Based on the prediction that the neural activity associated with the perception of an object’s shadow would be indicative of the neural activity of the body shadow, participants were immersed in a virtual environment and instructed to identify the ball shadow relative to the ball (BaSC) and their body shadow relative to their own position in space (BoSC). Data analysis included behavioral and electrophysiological measures.

At the behavioral level, the results indicated that the participants correctly identified the coincidence and non coincidence between the ball and the shadow (i.e., BaSC condition) and the body and its shadow (i.e., BoSC condition). They also revealed that their reaction times (i.e., RTs) were similar between coincident and non coincident sessions-during the decision making-in the BaSC condition and in the BoSC condition. In other words, immersed in a virtual reality environment, not only did the participants not experience motion sickness, but also were accurate and quick. At electrophysiological level, data analysis revealed that bilateral beta oscillations across anterior (frontal) and posterior (parietal and occipital) areas were similarly activated in “with” and “without” shadow sessions in both the BaSC condition and in BoSC conditions. It was also shown that bilateral beta frontal, parietal and occipital activations were not differentially involved when participants discerned the coincidence or non coincidence between the ball and its shadow (i.e., BaSC) on the one hand, and their body and its shadow (i.e., BoSC) on the other hand. At the behavioral and electrophysiological levels, under both coincidence and non coincidence situations, participants analyzed the shadow and the object (represented by a ball) in an identical manner. Similarly, they analyzed the shadow of the body, a singular object, in the same way as they analyzed their own body position in space (i.e., the physical body). Expressly, shadows are visual objects like any other type of object (Giannopulu et al., 2022b).

Based on the predictions formulated in the current study, the multiple regression analysis reported that increased beta oscillations in frontal, parietal, and occipital areas during ball shadow perception predicted increased frontal activity during body shadow perception. However, decreased beta oscillations in frontal, parietal, and occipital areas predicted decreased bilateral frontal activity during body shadow decision making. As such, the results are coherent with previous assertions according to which shadows are visual objects (Casati, 2012). They also enrich these assertions as it has been demonstrated that two kinds of shadow: geometric 3D shadows (i.e., spheric ball) and body shadows (i.e., human shaped) were analyzed as visual entities. The findings are also consistent with Kersten et al.’s (1996) data which displayed that shadows can afford relevant information about the object itself including the object’s motion, which corresponds to the ball and the body of the participant, in the present case. Overall, these findings illustrate that shadows are a reflection of objects and do not occur without objects (Mamassian, 2004; Casati, 2012). The present results extend this as it was demonstrated, for the first time, to the authors’ knowledge, that shadow affordance can also occur in 3D virtual environments, and this is analyzed as a full 3D perception of the ball and body in the virtual scene. The findings also revealed that the motion of the shadow relative to the motion of the ball does not induce illusory motion of the objects, even though the participants were immersed in a virtual environment conducive to inducing illusory behavior (Eskinazi and Giannopulu, 2021). Interestingly, such consequences are valuable for both ball and body shadows. At first glance, the results seem to be in contradiction to findings published by Kersten et al. (1996), which found that illusory motion of objects (i.e., apparent motion) can be induced from the motion of shadows. They also seem inconsistent with reports describing induction of illusory sensation of the whole body, i.e., “shadowed” changes in a patient’s body position triggered by electrical stimulation of the temporoparietal junction (Arzy et al., 2006). A possible interpretation of this lies in the fact that efficient perception of objects and shadows are the result of their mutual interaction, which seems to occur equally easily when objects and shadows have a consistent shape or are linked by a coincidental or not coincidental motion patterns. However, the methodological differences between the current study and the aforementioned studies do not really enable a direct comparison of the results. Essentially, the previous studies did not analyze ball and body shadows in the same population and 3D virtual environments and they did not consider neural electrophysiological components and behavioral components as was the case in the current study.

Consistent with Lovell et al. (2009), the current findings suggest that the ball and body shadows were both represented and unambiguously analyzed by the visual system. All participants were able to visually perceive the shadow (i.e., ball or body) and, depending on the condition, decide (i.e., clicking on key-response) when it was coincident or non coincident with the ball or their own body. In both coincidence and non coincidence conditions, the results indicate that participants exhibited similar reaction times in each experimental group individually, namely BaSC and BoSC. It seems that the visual system detects the coincidence or non coincidence between the shadow that the ball casts or the one that the body casts in 3D virtual environments without identification errors of illusory motion, that is, without anisotropy. That is to say, once immersed in the virtual environment, all participants were able to correctly perceive and report the relationship between each entity (i.e., ball and body) and its respective shadow. In other words, the coincidence or non coincidence of the ball shadow on the one hand and the body shadow on the other, with respect to the participant’s position in space, did not modulate the judged relationship between each visual entity and its respective shadow in the 3D virtual environment.

More than a peripheral visual analysis, that is, at retinal level, the beta oscillations of frontal, parietal and occipital neural activities bilaterally did not differ between coincident and non coincident sessions for either entity: the ball and its shadow and the body and its shadow. This suggests that the coincidence or non coincidence between entities and shadows was not represented in distinct areas of the brain, but that the representations of these entities and shadows would depend on environmental land marks and egocentric perception. Specifically, the results suggest activation of the occipito-parieto-frontal pathway which belongs to a distributed neural network and is involved in embodied actions (Tootell and Taylor, 1995). These results provide support for the assumption that the brain deduces information associated with the position of the visual entities (i.e., ball and the body and their shadows) from bodily signals (Paillard, 1991; Yamamoto et al., 2005; Arzy et al., 2006; Blanke, 2012; Riva, 2018). The findings also imply that the immersion into 3D virtual environments does not affect the brain’s inferential capacities for either entity (i.e., ball and body), and the shadows that they cast. With the above in mind, it appears that not only real but also virtual entities including their (virtual) shadows could modify cerebral representations and that the cerebral representations of these entities and the relationship they sustain with the body are constantly updated in virtual environments. The results can also be associated with recent data demonstrating that body shadow animations involve frontal neural activity of healthy participants (Alaerts et al., 2009) and improve the body representations in stroke patients (Russo et al., 2017). In both coincident and non coincident sessions and for both entities (i.e., ball and body and their shadows), participants reported only correct responses and exhibited similar anterior and posterior beta oscillations activities. The current findings seem consistent with the statement that objects and their shadows are incorporated into the body, they improve body representations and extent the body (Poirier and Hardy-Vallee, 2005; Kuylen et al., 2014). As such, these results also appear to support recent studies and more importantly, scientific speculations (Pavani and Castiello, 2004; Pavani and Galfano, 2007), which suggest the incorporation of objects and shadows and the resultant body extension not only occur in real but also virtual reality environments. Depending on the activation of distributed representations of visuospatial and sensorimotor information in the occipito-parieto-frontal pathways (Goodale, 2008), the findings support the consideration that the capacity to identify objects is driven by the sensorimotor experience people have with objects (e.g., ball and body in the current situation) and seem to be the case, in both real and virtual environments. Based on the similarities in brain activities it appears that perceptual processes in real and virtual environments are both “object-dependent” and “shadow-dependent.”

Considering that the internalization, emulation and simulation of shadows could serve as a predictive model that envisions the neural activity of the body shadow, the current results report that the bilateral frontal, parietal and occipital beta oscillations associated with the ball shadow might be an indicator of the bilateral frontal beta oscillations related to body shadow. Specifically, it appears that the body shadow perception was predicted by the object shadow perception. Bilateral frontal, parietal and occipital beta oscillations activity associated with the ball shadow likely preceded the body shadow and provided a direct measure of the frontal beta oscillations that correspond to the neurophysiological correlates of prediction. Body shadow perception would be “ball shadow-dependent.” This is not only consistent with existing data reporting that objects’ shadows are considered a continuation of the body and are inclined to create a sense of embodiment, i.e., they are part of ourselves (Kuylen et al., 2014), but also enriches these data suggesting that the embodied objects’ shadows significantly contribute to body representation and are used as a predictive reference for the body shadow. The body shadow would therefore be seen and understood as a substitute for the organic body, that is, the body shadow is a kind of vicarious body.

One may suggest that it is controversial whether the bilateral beta frontal oscillations are an authentic reflection of body shadow perception, i.e., that the cortical sources underlying the inference potential reflect the body shadow and its relationship with the participant position in space even before its presence. Nevertheless, it should be considered that the findings are consistent with several data according to which predictions associated with body representations involve frontal, parietal and occipital areas (Bubic et al., 2010). Contrary to the assumption that only motor areas (i.e., frontal areas) provide the basis for predictions (Pickering and Gambi, 2018), the current data signify advancement in aligning the brain motor (frontal) and sensory (parietal and occipital) correlates of predictions. As such, they provide relative support for the implication of motor areas in prediction, but also imply that predictive mechanisms involve multiple bilateral brain areas including motor regions. More importantly, such predictive mechanisms seem to exist in real (Pulvermüller and Grisoni, 2020) and in virtual environments as reported by the present study. Dynamic *per se*, occipito-parieto-frontal cortical sources underlying the potential of cortical predictive capacities mainly reflect perceptual and motor components of prospective future events before, they even occur. Sensory, motor and perceptual representations inherently generate probabilities, and draw and construct prospective abstract representations over attainable percepts. In essence, neural predictions along with inference and exploration would be a supplementary general principle of cortical function in real and virtual environments. As a whole, the above mentioned findings suggest that beta oscillations proceeded as an activator filter throughout the cortex, inferencing the location and likely timing of the body shadow, i.e., when and where it would occur. This is coherent with Sherman et al. (2016) data according to which beta motor and somatosensory coordinations mediate top-down predicted behavior. They also imply possible functional similarities between sensory, motor and cognitive beta oscillations. The above considerations could also account for the inferential mechanisms related to decision making process associated with body shadow.

The results demonstrated that beta oscillations of the bilateral occipito-parieto-frontal areas associated with ball shadow perception predicted a decrease in bilateral frontal neural activity at the beta band level related to the decision making process of the body shadow. Based on the function of beta oscillations, an additional explanation for this could be echoed at the decision making process itself. Attempting to analyze this leads to a consideration of the components involved in this decision making. In the current study, the participants were instructed to press the key-response to declare if the body shadow was in coincidence or non coincidence with their own position in 3D space. Assuming that the decision making process involves three main temporal components: sensory, decisional and motor, the sensory component would correspond to the onset of the visual information and the onset of neural activation of occipital, and parietal areas that specify perception go the entities (i.e., both ball and body shadows). The decisional component would coincide with the duration of time that elapsed between the occipito-parietal activation and the participant’s encoded decision and would involve premotor frontal activation that indicates preparation to undertake a judgment. The motor component would conform to the time necessary to produce a response after the decision and would be associated with motor frontal intervention. When beta oscillations were considered, it was suggested that their modifications before movement are associated with the framing and designing the movement goal (Schmidt et al., 2019). In other words, bilateral beta desynchronization signifies the transition between the moment of somatosensory perception and the motor decision. According to the current findings, bilateral occipito-parieto-frontal beta desynchronization associated with ball shadow perception predicted bilateral frontal beta desynchronization associated with the decision making of body shadow perception relative to the body position. Specifically, beta oscillatory modulation reduction in sensorimotor areas associated with ball shadow perception mirrored a reduction in beta oscillations in frontal areas corresponding to the body shadow perception. The results are coherent with existing data, showing that beta oscillatory modulations are interconnected with the information characteristics and the decision-making process (Herding et al., 2016; Spitzer and Haegens, 2017). They also suggest that bilateral beta oscillations of ball shadow perception would be reflective of the subsequent decision making of body shadow. Such findings could be interpreted in light of a supramodal framework in which bilateral beta oscillatory modulations would mirror the dynamic recruiting of the shadow-relevant neural network (i.e., both ball and body shadow). This is coherent with the flexible and transient mechanisms that underlies beta oscillations, which is reported to reflect functionally relevant representations, facilitate inter communication between networks (Siegel et al., 2011) and perceptual and motor top-down interactions (Sherman et al., 2016).

In summary, the current study identified similar beta oscillations in bilateral frontal, parietal and occipital brain areas between “with” and “without” shadow sessions and coincident and non coincident motion patterns within each BaSC and BoSC conditions independently were reported. The coincidence or non coincidence between the ball and its shadow and the body and its shadow did not affect reaction time behavior. In addition, it was found that body shadow specific beta oscillatory modulations in the bilateral frontal areas reflect ball shadow relevant sensorimotor perception, i.e., dorsal visual pathway, and subsequent decision making in 3D virtual environments. Such beta oscillatory modulations would be an expression of the formation of predictive neural frontal assemblies, which encode and infer body shadow neural representation, that is, a substitution of the physical body. These findings confirm already existing data on the way the brain harmonizes itself to situations and obtains information from objects and their shadows.

A potential limitation of the current study is that participants in both the BoSC and BaSC conditions made real-time decisions by pressing a button. This could have introduced a hand lateralization effect in the reaction time and noise to the beta oscillations, potentially biasing the data. No lateralization effects or irrelevant oscillations were observed. In the current predictive scenario, the face could be expected to be the most significant part of the body and treated differently, as reported by Kanwisher and Yovel (2006), compared to the body itself, as described by Peelen and Downing (2005). However, it is important to note that in the present virtual reality (VR) shadow scenario, the face was considered as integral part of the body and was not distinguishable. Participants were unable to identify their own face when judging the coincidence or non coincidence between the object and its shadow, as well as the body or its shadow. Furthermore, the studies conducted by Peelen and Downing (2005) and Kanwisher and Yovel (2006) did not include shadows or utilize virtual reality environments in their experimental design. Additionally, they did not analyze the predictable relationships between brain activity. Although methodological differences prevent direct comparisons, it can be argued that the present study and the aforementioned ones are consistent in demonstrating the involvement of distinct brain regions in the processing of objects and bodies. Beyond this, our study suggests that distinct brain areas are activated not only by objects and bodies but also by their corresponding shadows, which appear to be inherently predictable.

Notwithstanding, the current findings prolong the existing data to the supra modal process by demonstrating that predictions are not exclusive to motor processing, but also to somatosensory and sensorimotor areas bilaterally. Furthermore, the current data suggest the existence of a cortical neural network in which the beta oscillatory dynamics of object shadows provide a mechanism for the formation of functional networks during the internal re/activation of body relevant cortical representations. As such, it can be suggested that prediction along with inference and exploration are general principles of cortical functioning in real and 3D virtual environments.

## Data availability statement

The participants did not provide consent for their data to be utilised for different purposes for those described in the original study aims and therefore the datasets cannot be made publicly available. Although de-identified versions of the datasets used for the current study may be made available on reasonable request.

## Ethics statement

The studies involving humans were approved by Bond University Human Research Ethics Committee (BUHREC 16121). The studies were conducted in accordance with the local legislation and institutional requirements. The participants provided their written informed consent to participate in this study.

## Author contributions

IG: conceptualization, creation, methodology, data collection, formal analysis, supervision, investigation, and writing – original draft. GB: formal analysis, data, and graphic curation. KL and EA: virtual environment development. AN-H: MatLab support. MV, TL, JG, PG, and GI: contribution to data collection. All authors contributed to the article and approved the submitted version.
